# Pain in Parkinson´s Disease: A Cross-Sectional Study of Its Prevalence, Types, and Relationship to Depression and Quality of Life

**DOI:** 10.1371/journal.pone.0136541

**Published:** 2015-08-26

**Authors:** Peter Valkovic, Michal Minar, Helena Singliarova, Jan Harsany, Marta Hanakova, Jana Martinkova, Jan Benetin

**Affiliations:** 1 Second Department of Neurology, Faculty of Medicine, Comenius University, Bratislava, Slovak Republic; 2 Laboratory of Motor Control, Institute of Normal and Pathological Physiology, Slovak Academy of Sciences, Bratislava, Slovak Republic; 3 Department of Physiatry and Rehabilitation, Ruzinov University Hospital, Bratislava, Slovak Republic; 4 Department of Neurology, Faculty of Medicine, Slovak Medical University, Bratislava, Slovak Republic; University of Tennessee Health Science Center, UNITED STATES

## Abstract

Pain is an important and distressing symptom in Parkinson’s disease (PD). Our aim was to determine the prevalence of pain, its various types and characteristics, as well as its impact on depression and quality of life (QoL) in patients with PD. How pain differs in early- and advanced-stage PD and male and female PD patients was of special interest. One hundred PD patients on dopaminergic medications had a neurological examination and participated in a structured interview on pain characteristics and completed standardized questionnaires. A total of 76% of the patients had pain. The following types of pain were present: musculoskeletal pain accounted for 41% of the total pain, dystonic pain for 17%, central neuropathic pain for 22%, radicular pain for 27%, and other pains (non-radicular low back pain, arthritic, and visceral pain) made up 24%. One type of pain affected 29% of all the subjects, two types 35%, three types 10%, and four types of pain were reported by 2%. All types of pain were more prevalent in advanced-stage PD subjects than in early-stage PD subjects, except for arthritic pain (subclassified under”other pain”). The frequency and intensity of actual, average, and worst experienced pain were significantly more severe in advanced-stage subjects. PD subjects with general pain and in advanced stages were more depressed and had poorer QoL. Depression correlated with worst pain in the last 24 hours and with pain periodicity (the worst depression score in patients with constant pain). QoL correlated with average pain in the last 7 days. Pain is a frequent problem in PD patients, and it worsens during the course of the disease.

## Introduction

Parkinson’s disease (PD) is a complex disorder characterized by various motor, sensory, autonomic, and psychiatric signs and symptoms [1]. Pain is a frequent complaint of many PD patients, occurring in 30–85% of subjects (mean = 66%). Table 1 lists the main findings of a few frequently cited studies on the prevalence of pain in PD. The obviously discrepant findings are a result of the differences in sample sizes, scales, and classifications of types of pain [2–11].

**Table 1:** Overview of studies on the prevalence of pain in Parkinson´s disease.

Reference	N	Prevalence	Pain classification	Relationship to other domains assessed
[2]	95	46%	Musculoskeletal	Correlation with lower age
			Dystonia	
			Radicular/neuritic	
			Akathisia	
			Joint	
[3]	117	40%	Dystonic	Correlation with UPDRS IV
			Non-dystonic	
[4]	450	67%	Related to PD	Correlation with depression
			Not related to PD	
[5]	96	64.9%	Musculoskeletal	No correlation with depression
			Radicular or neuropathic	
			Secondary to dystonia	
			Central	
[6]	121	66%	Dystonic	
			Paresthesia/neuropathic	
			Musculoskeletal	
[7]	402	69.9%	Dystonic	
			Arthralgic	
			Cramping	
			Peripheral neuropathic	
			Central neuropathic	
[8]	176	83%		
[9]	50	56%	Musculoskeletal	
			Dystonic	
			Radicular	
			Articular	
			Headache	
			Unspecified	
[10]	901	29.9%		
[11]	123	85%	PD-related	
			Unrelated to PD	
			Indirectly related to PD	
			Multiple causes	
			Treatment related	

There are various classification systems of pain for PD. For example, Deuschl and Wassner [12] recommended that pain be divided into nociceptive (musculoskeletal, visceral, cutaneous) and neuropathic (peripheral, central) types. In contrast, Chaudhuri and Schapira [13] distinguished musculoskeletal, PD-related chronic pain, fluctuation-related, nocturnal, coat-hanger, orofacial, peripheral limb and abdominal pain. Ford’s pain classification from 2010 [14] remains the most-cited one. It distinguishes among five crucial types of pain in PD—according to their origin, as well as the treatment approach (Table 2).

**Table 2:** Ford’s pain classification.

Pain type	Pain features
Musculoskeletal pain	Aching, cramping, arthralgic, myalgic sensations in joints, and muscles
	Associated findings may include muscle tenderness, arthritic changes, skeletal deformity, limited joint mobility, postural abnormalities, and antalgic gait
	May be exacerbated by parkinsonian rigidity, stiffness, and immobility, and relieved by mobility
	May fluctuate with dose of medication and improves with levodopa
Radicular/neuropathic pain	Pain in a root or nerve territory, associated with motor or sensory signs of nerve or root entrapment
Dystonic pain	Associated with sustained twisting movements and postures; muscular contractions often very forceful and painful
	May fluctuate closely with medication dosing: early morning dystonia, off dystonia, beginning-of-dose and end-of-dose dystonia, peak dose dystonia
Central or primary pain	Burning, tingling, formication, “neuropathic” sensations, often relentless and bizarre in quality, not confined to root or nerve territory
	Pain may have an autonomic character, with visceral sensations or dyspnea, and vary in parallel with the medication cycle as a non-motor fluctuation
	Not explained by rigidity, dystonia, musculoskeletal or internal lesion
Other pain	Primary headache, visceral, arthritic, non-radicular low back pain, oral and genital pain

**Note:** in an original paper Ford proposed a 5^th^ type of pain: “akathitic discomfort” [14]; we substituted this type in our study by category “other pain”.

Chronic pain has shown a clear correlation with severity of depression and reduced quality of life (QoL) in the general population [15, 16]. However, little is known about the particular association of pain in PD with depression and QoL. Only a few recent studies have examined the impact of pain on mood and health-related QoL in PD. Their findings have been contradictory [17–20].

The aim of this study was to determine the prevalence of pain, its various types and characteristics, and its impact on depression and quality of life in PD patients. We were especially interested in elucidating the differences in pain between early-stage and advance-stage as well as male and female PD subjects.

## Materials and Methods

### Subjects

One hundred consecutive subjects (50 men; 50 women) with a diagnosis of idiopathic PD according to the UK Parkinson's Disease Society Brain Bank criteria [21] participated in the study. Informed consent was obtained in accordance with the Declaration of Helsinki, and the local Ethics Committee (Academic Derer´s University Hospital, Bratislava) approved the study protocol. Patients with mild cognitive impairment were included provided informed written consent could be obtained, and they were able to complete clinimetric questionnaires alone or with the help of their caregiver. We excluded subjects with diagnosed dementia. The subjects had the full capacity to consent because they maintained general cognitive function and daily activities. We included only patients who had been fully able to understand and cooperate with study procedures. There was not any surrogate consent procedure consented on the behalf of participants. All patients have been on dopaminergic therapy with levodopa (plus dopa-decarboxylase inhibitor) and/or dopamine agonists for at least 3 months. Untreated subjects or those on advanced therapies (deep brain stimulation, subcutaneous apomorphine pump, or levodopa/carbidopa intestinal gel) were not included.

The patients were divided into an “early-stage” (ES) and an “advanced-stage” (AS) group on the basis of the modified Hoehn and Yahr score (H&Y) [22], the cut-off of 2.5, and a history of late complications of levodopa therapy. Six patients were classified as H&Y1, one as H&Y1.5, thirty-one as H&Y2, and nine as H&Y2.5 (ES subjects). Forty-five people were in H&Y stage 3, and eight in H&Y4 (AS patients). Dopaminergic therapy load was expressed in terms of levodopa equivalent daily dose (LEDD) [23]. Each subject was in the state of best possible control of parkinsonian motor symptoms during the assessment. Fluctuating patients were assessed in their optimal “on” state. Demographic and basic clinical data are presented in Table 3.

**Table 3:** Demographic and basic clinical data.

	Altogether (N = 100)	Early stages (N = 47)	Advanced stages (N = 53)	P-value
**Sex (male:female)**	50: 50	26: 21	24: 29	
**Age (years)± SD; (range)**	65.5 ± 8.8	62.9 ± 8.0	67.9±8.9	NS
	(47–85)	(47–76)	(47–85)	
**Disease duration (years)± SD; (range)**	5.9 ± 4.4	4.1±3.4	7.5±4.6	0.004
	(0.5–19)	(0.5–14)	(0.5–19)	
**L-dopa equivalent daily dose (mg)± SD; (range)**	935.5 ± 476.6	702.1 ± 337.0	1142.5 ± 489.1	<0.001
	(100–2000)	(100–1500)	(300–2000)	
**Hoehn & Yahr score; (range)**	2.6 ± 0.7	2.0±0.4	3.2±0.4	<0.001
	(1–5)	(1–2.5)	(3–4)	

### Methods

Validated Slovak translations of the following screening and diagnostic instruments were applied:

- The Brief Pain Inventory (BPI); a self-report questionnaire using a 10-point Likert scale as a response alternative assesses pain intensity and interference with functions [24]. The variable of interest in this study was the pain severity index, calculated by adding the scores on the pain severity items (worst pain, least pain, and average pain in the last 24 hours, pain now, and average pain in the last week) [8].
- The Leeds assessment of neuropathic symptoms and signs (LANSS) Pain Scale [25], a cut-off score for the presence of neuropathic pain, was 12; the maximum score was 24.
- The Parkinson's disease questionnaire with eight dimensions (PDQ-8); health-related quality of life measure [26]; a summary index (PDQ-8 SI) was used for statistical analysis
- The Beck Depression Inventory-II (BDI); a 21-question, multiple-choice self-report inventory for measuring the severity of depression [27]

PDQ-8 was completed by patients (with the aid of caregivers if necessary) while waiting to be seen by a neurologist. The BDI, BPI, and LANSS were administered by a trained investigator.

The frequency, duration, and development were assessed for each type of pain. Also the patients’ perception of the pain as dependent on motor fluctuations was determined. Finally, the patients were asked how the dopaminergic medication influenced the pain and if they perceived the pain as directly related to their PD. Patients were asked about the position, radiation, onset, periodicity (i.e., constant or intermittent), character, associated symptoms, precipitating and relieving factors of each pain. Analgesic use was also determined but was not statistically evaluated.

For the review of pain in PD, a literature search was undertaken using PubMed database and relevant search terms. Articles were screened for suitability and data relevance.

### Statistical analysis

Statistical examination of the data was performed using IBM SPSS Statistics 20. Clinical and demographic variables of both groups of PD patients were compared by Student’s T-test for parametric data, and by the Mann-Whitney U-test for non-parametric variables (*P* ≤ 0.05 was considered to be significant). As the Kolmogorov—Smirnov test showed that not all data were normally distributed, Spearman’s rank correlation coefficient (Rho) was used to evaluate the association between the measured parameters (*P* ≤ 0.01 was considered to be significant for correlations).

## Results

**Fig 1:**
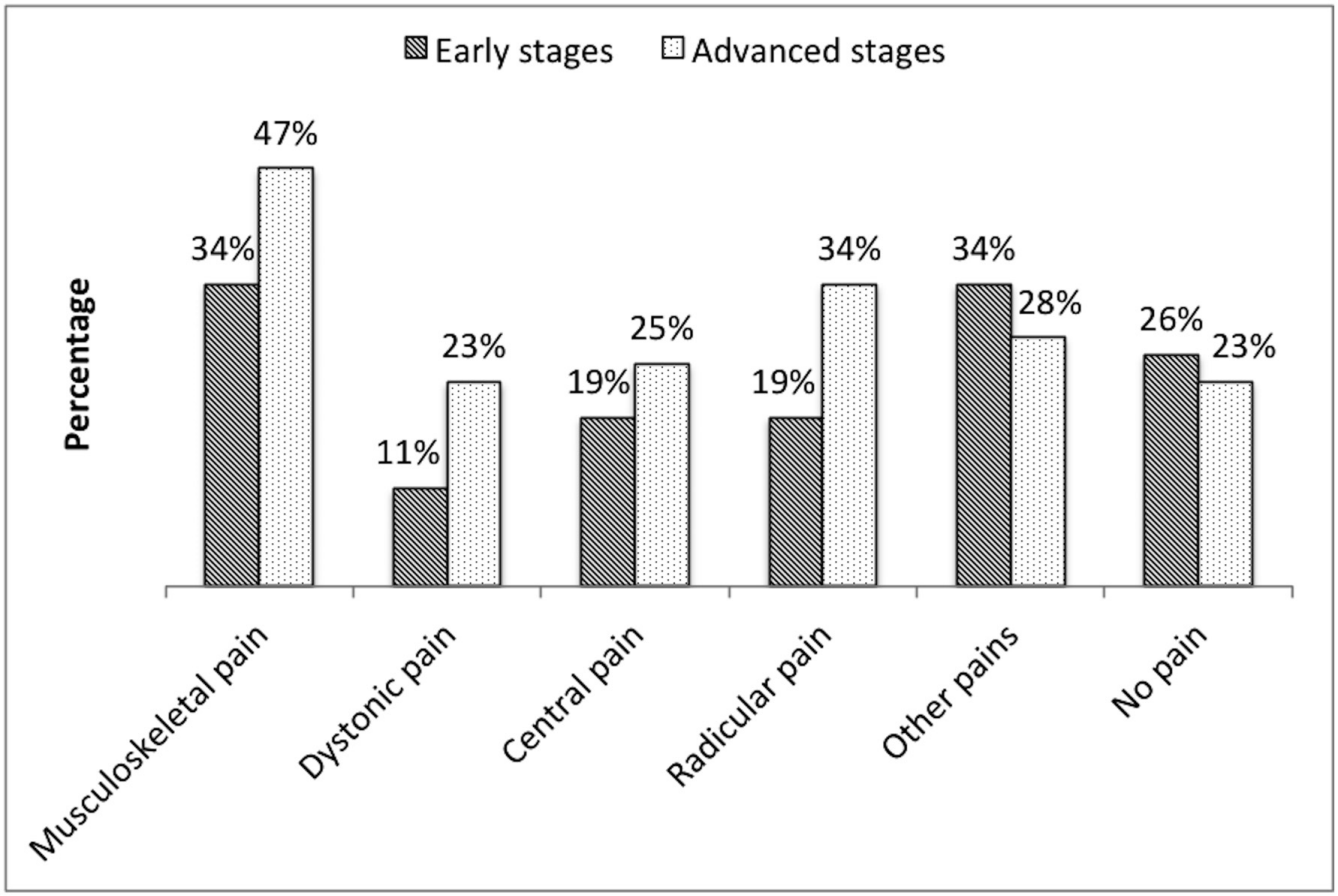
Distribution of pain types in PD patients. Note the higher prevalence of all types of pain except for “other pain” in the advanced stage of PD.

No pain was reported by 24% of all 100 examined subjects; 76% reported some type of pain in the last week. The occurrence of the types of pain among the subjects was as follows: 41% had musculoskeletal pain, 27% radicular pain, 22% central pain, 17% dystonic pain, and 31% other types of pain (headache, visceral, athritic, nonradicular low back pain). The differences in distribution of pain types according to early and advanced stages of PD are shown in Fig 1. The most frequent pain in the category “other pain” was non-radicular low back pain (ES 17%; AS 19%; 18% altogether), followed by arthritic pain (ES 15%; AS 8%; 11% altogether), and visceral pain (ES 2%; AS 2%; 2% altogether). No patients reported primary headache or oral and genital pain.

**Fig 2:**
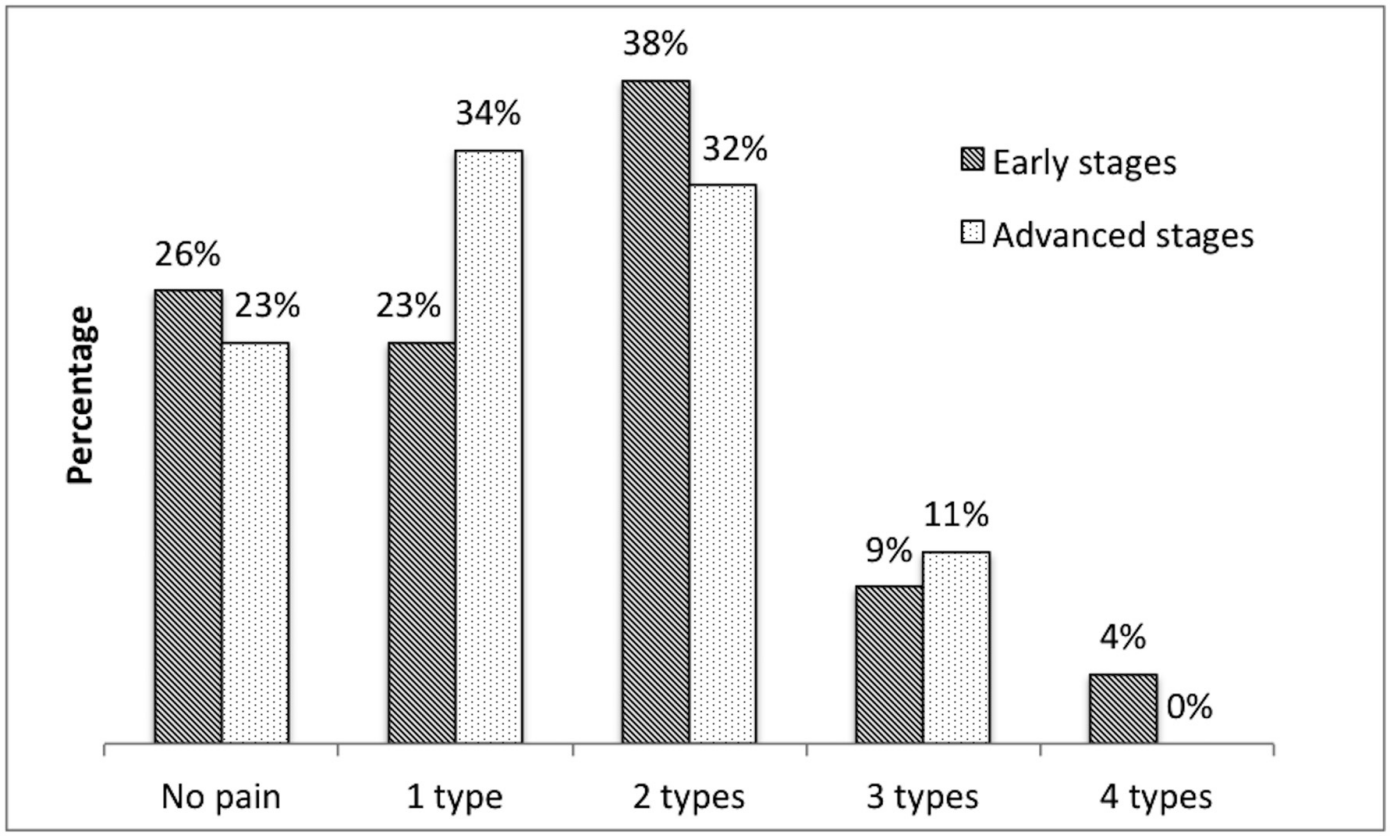
Types of pain in PD patients.

A total of 29% of the entire set of subjects suffered from one type, 35% from two types, 10% from three types, and 2% from four types of pain (Fig 2).

The number of types of pain correlated positively with pain periodicity (short-lasting, intermittent, constant) (Rho = 0.401); the more types of pain, the more constant was the complaint.

A comparison of the parameters by the stage of PD revealed that the incidence of dystonic pain was significantly higher in advanced PD. Actual, average, and worst intensities of pain were significantly more severe in the advanced stage. Overall scores of BDI and PDQ-8 were significantly higher in the advanced stage (Table 4).

**Table 4:** Comparison of early and advanced stages of PD.

Parameter	Early stage	Advanced stage	p value
Pain average/ 7 days	3.1±2.3	3.8 ± 2.6	**0.02**
Pain worst/24 h	2.6±2.6	3.8 ± 2.8	**< 0.001**
Pain average/24 h	2.3±2.0	2.9 ± 2.0	**0.01**
Pain now	1.3±1.7	2.2±2.8	**0.03**
BDI score	7.0±4.8	11.5±5.6	**< 0.001**
PDQ-8 score	11.3±8.5	18.1±9.5	**< 0.001**
Number of pain types	1.3±1.0	1.4±1.1	0.07

Significant results are bold.

If subjects were compared according to the presence or absence of pain, those suffering from any type of pain had a significantly more severe score in PDQ-8 (10.1 ± 5.3 vs. 7.0 ± 6.4; p = .02), and BDI (16.6 ± 9.4 vs. 9.7 ± 7.6; p <0.001). Differences in the measured parameters between men and women are shown in Table 5. Correlations of pain characteristics with quality of life, depression, and selected variables of interest are given in Table 6.

**Table 5:** Comparison of the pain characteristics between the sexes (variables with significant differences).

	Mean values (men; women)	P-value	Preponderance
**Age**	62.96; 68.12	<0.001	Women
**Disease duration**	4.86; 6.98	0.01	Women
**Pain now**	1.36; 2.18	0.05	Women
**Pain average—last week**	2.84; 4.10	0.02	Women
**Pain worst—last 24 hours**	2.56; 3.92	0.02	Women

**Table 6:** Correlations of pain characteristics expressed as Spearman’s rank correlation coefficient with quality of life, depression, and selected variables of interest.

	Pain average/ 7 days	Pain worst/ 24 h	Pain least/ 24 h	Pain average/24 h	Pain now	Number of pain types	Pain periodicity
**PDQ-8-SI**	**0.26**	NS	NS	NS	NS	NS	NS
**BDI**	NS	**0.31**	NS	NS	NS	NS	**0.32**
**Age**	NS	NS	NS	NS	NS	NS	NS
**Disease duration**	NS	**0.28**	NS	**0.30**	**0.30**	NS	NS
**L-dopa equivalent daily dose**	NS	NS	NS	NS	NS	NS	NS
**Hoehn & Yahr score**	NS	**0.27**	**0.30**	NS	NS	NS	NS

Correlation is significant at the P ≤ 0.01 level (2-tailed); P values are not shown.

## Discussion

### Prevalence and types of pain

In this cross-sectional study of pain in PD we found that 74% of PD subjects on dopaminergic medication had experienced some type of pain. Musculoskeletal pain (41%) and radicular back pain (27%) were the most prevalent types. Dystonic pain (17%) was the least prevalent type. Dystonic and musculoskeletal pains were by definition alleviated by dopaminergic medication. Quinn et al [28] stressed the role of dopaminergic treatment in severity of pain in PD patients. The fact that acute administration of levodopa raises the pain threshold in subjects with PD confirms that the dopaminergic system is involved in sensory processing [29]. Treatment with levodopa also leads to the induction of motor fluctuations (off-dystonia, “peak dose”, and/or biphasic dyskinesias) resulting in worsening of dystonic/dyskinetic pain. The prevalence of these three pain types varies from study to study, depending on the primary focus of the study. For example, Beiske et al. [8] reported 70% prevalence of musculoskeletal, 40% dystonic, and 20% radicular pain in their parkinson cohort. In contrast, Broetz et al. found up to 36% occurrence of radicular pain [30]. Rana et al. [6] observed that musculoskeletal pain was prevalent only in 28% and dystonic pain, in 48%. Our findings showed the highest prevalence (22%) for central neuropathic pain, in contrast to previous studies (4.5–12.7%) [5, 7, 8].

Limitation of our study could be the fact that we did not assess the influence of analgesic medication on prevalence of pain. It affects mostly the intensity of pain, the occurrence itself and the pain frequency is less dependent.

The above-mentioned differences may have several explanations, such as different severity stages in the studied PD populations, methods of classification used, or primary outcomes of interest. We tried to avoid discrepancies due to the confounding effect of testing patients in any medication state as this could affect their mood and pain threshold [31, 32]. For this reason we assessed our patients in their “best-ON” medication state.

Twenty-nine percent of our PD population suffered from one type, 35% from two types, and 10% of subjects reported three types of pain. Interestingly two subjects (2%) reported having four concurrent types of pain. This is a very important finding, because each type of pain requires a different therapeutic approach (for review see [6]).

### Early versus Advanced Stages of Parkinson´s Disease

All pain types were more prevalent in patients with advanced-stage PD than in early stages. Only arthritic pain—classified under “other pain“—was more prevalent in patients with early-stage PD. The frequency and intensity of actual, average, and worst experienced pain were as expected significantly more severe in patients with advanced-stage PD.

Musculoskeletal, radicular, and dystonic pain become more frequent as the disease progresses. This is probably due to long-term pathologically increased muscle tone and the worsening of postural reflexes. Moreover, as these postural disorders (anteflexion, kyphoscoliosis) progress, a non-radicular low back pain gradually converts into radicular pain [30]. Central pain probably deteriorates along with neurodegeneration, which spreads to the central sensory pathways [14].

Our exclusion criteria had eliminated any patients on advanced continuous forms of therapy (deep brain stimulation, levodopa/carbidopa intestinal gel, subcutaneous apomorphine) which significantly reduce fluctuations (together with dystonic/dyskinetic pain). On the one hand, this caused our population to be more homogeneous and led to more consistent data. On the other, this was a limitation since the subgroup of patients was excluded that comprises a significant population of PD patients in most movement disorder centres.

### Men vs. women

While several studies found no difference in pain prevalence [3–6] between men and women with PD, others [8, 33] reported that female patients suffered significantly more from pain than male PD patients. Not only social conditioning and psychosocial factors influence sex differences in pain perception, but sex hormones and different endogenous opioid systems may also play a role [34]. Our results are congruent with the latter statement—i.e., female PD patients experienced pain sensations more intensely than male PD patients and three pain subscores were significantly higher in women. Compared to men, they perceived actual pain, worst pain in the last 24 hours, as well as average pain in the last week significantly more severely. This is in line with findings that women assimilate pain in a more intense manner and report pain more frequently than men [35].

The women were significantly older with higher median duration of disease compared to men. Since disease duration correlates with pain intensity and/or frequency, it is difficult to confirm if the higher prevalence of pain is related to the gender. Disease duration and progression seem to be the crucial factor in pain severity.

### Impact on depression and quality of life

A comparison of subjects according to the presence of pain revealed that those with any type of pain had a significantly more severe score in PDQ-8 and in BDI. Moreover, the overall scores in BDI and PDQ-8 were significantly higher in advanced stages than in the early stage. Depression correlated with worst pain in the last 24 hours and with pain periodicity (the worst depressive score in patients with constant pain). Quality of life correlated with average pain in the last 7 days. Although these correlations were statistically significant, the strength of significance was low. The correlation studies of PD-related pain and depression mentioned earlier yielded contradictory results, suggesting that pain in PD is a relatively independent phenomenon and its impact on depression and quality of life is hard to be quantified.

In light of the major motor symptoms of PD and the efficacy of dopaminergic treatment, PD used to be considered a disorder of the dopaminergic system. Recent data provide a broader perspective on the pathophysiology of PD—most of the treatment-resistant symptoms of advanced PD (postural instability, cognitive decline, depression, autonomous dysfunction) are attributed to an imbalance in cholinergic, serotonergic, glutamatergic and noradrenergic neurotransmission [36, 37]. This is in line with the absence of a correlation of pain with dopaminergic load (calculated as L-dopa equivalent daily dose) reported in our study. Albeit sometimes partly responsive to L-dopa, central pain could be one of the symptoms caused by a disturbance in non-dopaminergic neurotransmission. A study that assessed pain sensitivity after STN-DBS observed that the treatment had had no significant effect, thus supporting this view [38]. In addition, Dellapina et el. showed with PET neuroimaging that the dopaminergic system would probably not be directly involved in pain in PD patients and that L-dopa could exert its antinociceptive effect by acting via noradrenergic and/or serotoninergic systems [31].

## Conclusions

Pain is a frequent problem in PD patients but it worasens during the course of the disease. Those PD patients who have pain are more depressed and have a poorer quality of life than those who do not. The prerequisites for optimal treatment of these patients are a correct diagnosis and classification of pain, since different therapeutic approaches are needed for different types of pain.
